# Oral probiotics to reduce vaginal group B streptococcal colonization in late pregnancy

**DOI:** 10.1038/s41598-020-76896-4

**Published:** 2020-11-12

**Authors:** Alex Farr, Valentina Sustr, Herbert Kiss, Ingo Rosicky, Alexandra Graf, Athanasios Makristathis, Philipp Foessleitner, Ljubomir Petricevic

**Affiliations:** 1 Department of Obstetrics and Gynecology, Division of Obstetrics and Feto-Maternal Medicine, Medical University of Vienna, Waehringer Guertel 18-20, 1090 Vienna, Austria; 2 Center for Medical Statistics, Informatics and Intelligent Systems (IMS), Medical University of Vienna, Vienna, Austria; 3 Department of Laboratory Medicine, Division of Clinical Microbiology, Medical University of Vienna, Vienna, Austria

**Keywords:** Microbiology, Applied microbiology, Clinical microbiology, Risk factors, Disease prevention, Neonatology, Preterm birth

## Abstract

This study aimed to evaluate the potential of oral probiotics to eradicate vaginal GBS colonization during the third trimester of pregnancy. We screened 1058 women for GBS colonization at 33–37 gestational weeks using a combination of vaginal-to-rectal swab and culture-based methods. Women who tested GBS positive were randomized to either the verum group, receiving a dietary probiotic supplement of four viable strains of *Lactobacillus* twice-daily for 14 days, or to the placebo group. Women underwent follow-up smears, whereat GBS colonization upon follow-up was considered the primary endpoint. We found that 215 women (20.3%) were positive for GBS upon screening, of which 82 (38.1%) were eligible for study inclusion; 41 (50%) of these were randomized to the verum and placebo groups each. After treatment, 21/33 (63.6%) members of the verum group, and 21/27 (77.8%) of the placebo group were still GBS positive (*p* = 0.24). Four (9.8%) women in the verum group and one (2.4%) in the placebo group experienced preterm birth (*p* = 0.20); smokers showed significantly higher rates of preterm birth (*p* = 0.03). Hence, the findings did not support the hypothesis that oral probiotics can eradicate GBS during pregnancy, although we observed a trend toward reduced GBS persistence after probiotic intake.

## Introduction

Group B Streptococcus (GBS), or *Streptococcus agalactiae*, is a Gram-positive bacterium that belongs to the group B of Lancefield that has shown a colonization rate of 10–30% in the vagina or rectum of pregnant women^1^. This colonization may be transient, intermittent, or persistent, and it is usually asymptomatic; differences in colonization rates may occur due to age, ethnicity, examined locations and microbiological processes^2^. Regardless of the planned mode of birth, ACOG guidelines recommend antenatal screening for vaginal GBS colonization, unless intrapartum antibiotic prophylaxis (IAP) is indicated due to GBS bacteriuria during pregnancy or there is a history of a previous GBS-infected newborn^3^. GBS is a potentially hazardous pathogen because of the potential for vertical transmission with subsequent neonatal sepsis, pneumonia, and meningitis, and it is defined by the time of occurrence as early- or late-onset neonatal GBS infection^4^.

Apart from GBS, the urogenital microflora of healthy pregnant women comprises a wide variety of microorganisms that differs in composition depending on exposure to several factors. The urogenital microflora is dominated by various *Lactobacillus* spp., which play an essential role in protecting women from genital infections^5^. A deficiency in lactobacilli can upset the microbial balance in the vagina and may result in increased susceptibility to colonization by pathogens, such as GBS^6^, as well as an increased risk of impaired pregnancy outcomes and preterm birth^7^. The supplementation of probiotics that contain *Lactobacillus* strains has been proven to improve cure rates and prevent infections^8^. *Lactobacillus* strains can disrupt the biofilm of pathogens, as it has been demonstrated *in-vitro* with *Gardnerella vaginalis*, thereby inhibiting pathogen growth^9^. Furthermore, there is some evidence that imbalances in the vaginal microbiologic milieu may increase the growth of mixed anaerobic bacteria^10^.

As there are preclinical data that support a positive effect on GBS colonization from administration of *Lactobacillus* spp., the current study aimed to evaluate the effects of oral intake of *Lactobacillus*-containing probiotics *in-vivo* on vaginal GBS colonization during late pregnancy. This could help to render IAP unnecessary, thereby preventing antibiotic resistance and reducing neonatal morbidity and mortality due to early- and/or late-onset neonatal GBS sepsis.

## Results

**Figure 1:**
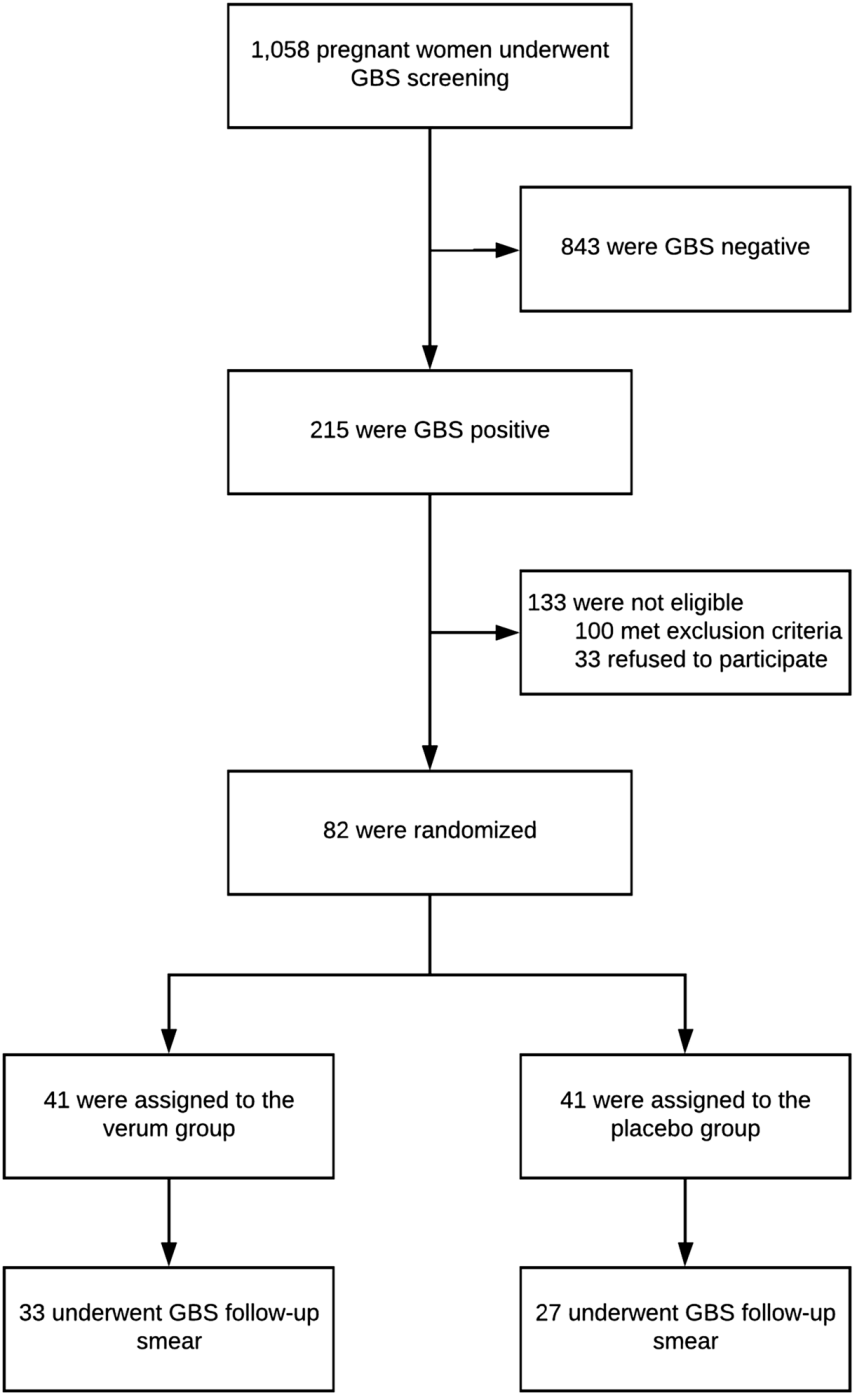
Screening, randomization, and follow-up of 1058 pregnant women.

A total of 215 (20.3%) of 1058 pregnant women, screened for GBS colonization, were positive, of which 82 (38.1%) were eligible for study inclusion (Fig. 1). Study participants were randomized into two groups (verum or control) of 41 women each (Table 1). After randomization, 22 of the 82 participants (36.7%) were dropped from the study for the following reasons: birth before scheduled follow-up smear (N = 16, 72.7%), refusal of follow-up smear (N = 3, 13.6%), prelabor rupture of membranes (N = 2, 9.1%), or inadequate intake of medication (N = 1, 4.6%). Consequently, the primary outcome was available for 33 women (80.5%) in the verum group, and 27 women (65.9%) in the placebo group. No adverse events were reported in any of the study groups.

**Table 1:** Maternal characteristics of 82 randomized study participants.

Variable	Verum group (N = 41)	Placebo group (N = 41)	*p*
	N (%) Mean ± SD Median (Min–Max)	N (%) Mean ± SD Median (Min–Max)	
**Gravidity**	2 (1–6)	3 (1–6)	0.50
**Parity**	2 (1–6)	2 (1–5)	0.51
**Maternal age**	35.1 ± 4.9	32.9 ± 4.6	0.03
**Smoking**			0.31
Yes	7 (17.1)	3 (7.3)	
No	33 (80.5)	36 (87.8)	
N/A	1 (2.4)	2 (4.9)	
**Previous preterm delivery**			0.49
Yes	0 (0)	1 (2.4)	
No	40 (97.6)	38 (92.7)	
N/A	1 (2.4)	2 (4.9)	
**Vaginal flora**^†^			0.74
Normal	35 (85.4)	37 (90.2)	
Intermediate	6 (14.6)	4 (9.8)	
**Vaginal candidosis**			0.49
Yes	2 (4.9)	0 (0)	
No	39 (95.1)	41 (100)	

N/A, not available; ^†^Patients with bacterial vaginosis were excluded from the study.

Among the 33 women in the verum group with available follow-up smears, 21 (63.6%) were GBS positive at follow-up, compared to 21 of 27 women (77.8%) in the placebo group. This difference was not statistically significant upon logistic regression analysis (*p* = 0.24). In addition, Nugent scoring of the vaginal flora on Gram-stained smears was not significantly different between the study groups (*p* = 0.44). Smoking status (*p* = 0.93), maternal age (*p* = 0.41), and vaginal candidosis (*p* = 0.50) did not have an impact on GBS colonization upon follow-up. Odds ratios and confidence intervals are provided in Table 2.

**Table 2:** Regression models for primary and secondary endpoints.

Variable	Odds ratio estimate	95% Confidence interval	*p*
**GBS colonization at follow-up**			
Study group (verum vs. placebo)	0.500	0.158–1.582	0.24
Smoking status	1.081	0.189–6.169	0.93
Maternal age	1.049	0.936–1.175	0.41
Vaginal candidosis	0.375	0.022–6.384	0.50
**Preterm birth**			
Study group (verum vs. placebo)	0.833	0.254–2.732	0.76
Smoking status	1.667	0.304–9.128	0.56
Parity	0.994	0.600–1.648	0.98
Maternal age	1.032	0.913–1.167	0.61
**Birthweight**			
Study group (verum vs. placebo)	− 45.390	248.062–157.282	0.66
Smoking status	− 282.414	582.216–17.387	0.07
Parity	24.938	− 55.482–105.358	0.55
Maternal age	0.858	− 20.125–21.841	0.94
**Gestational age at delivery**			
Study group (verum vs. placebo)	− 0.171	− 0.774–0.432	0.58
Smoking status	− 0.583	− 1.415–0.250	0.17
Parity	0.019	− 0.203–0.240	0.87
Maternal age	− 0.035	− 0.097–0.027	0.27
**Mode of delivery**			
Study group (verum vs. placebo)	1.000	0.420–2.382	1.00
Smoking status	2.139	0.511–8.961	0.30
Parity	0.794	0.555–1.135	0.21
Maternal age	0.974	0.890–1.066	0.57

With regard to the secondary outcome parameters, four women (9.8%) in the verum group and one (2.4%) in the placebo group experienced preterm birth, a difference that was not statistically significant (*p* = 0.20; Table 3). In the univariate analysis, a significant difference in the occurrence of preterm birth was found between smokers and non-smokers (*p* = 0.03), but not for the other potentially confounding factors of parity (*p* = 0.84) and maternal age (*p* = 0.12).

**Table 3:** Perinatal outcomes of 82 randomized study participants.

Variable	Verum group (N = 41)	Placebo group (N = 41)	*p*
	N (%) Mean ± SD Median (Min–Max)	N (%) Mean ± SD Median (Min–Max)	
Gestational age at delivery	38.5 ± 1.4	38.6 ± 1.2	0.58
Neonatal birthweight	3359 ± 461	3404 ± 475	0.66
Birthweight percentile	47.2 ± 27.4	48.1 ± 28.4	0.88
Apgar score at 1 min	9 (7–10)	9 (3–9)	0.23
Apgar score at 5 min	10 (8–10)	10 (6–10)	0.89
Apgar score at 10 min	10 (9–10)	10 (8–10)	0.49
Umbilical cord pH	7.26 ± 0.08	7.25 ± 0.08	0.88
Preterm delivery			0.20
Yes	4 (9.8)	1 (2.4)	
No	37 (90.2)	40 (97.6)	
PROM			1.00
Yes	6 (14.6)	7 (17.1)	
No	35 (85.4)	34 (82.9)	
Mode of delivery			1.00
Vaginal	18 (43.9)	18 (43.9)	
Instrumental	1 (2.4)	1 (2.4)	
C-section	22 (53.7)	22 (53.7)	
Neonatal sepsis			1.00
Yes	0 (0)	0 (0)	
No	38 (92.7)	41 (100)	
N/A	3 (7.3)	0 (0)	

N/A, not available; PROM, prelabor rupture of membranes.

The secondary outcome parameters gestational age at delivery, neonatal birth weight, live birth, and mode of delivery were not statistically significantly different between the verum and placebo group. Perinatal outcomes of the 82 randomized participants are shown in Table 3. No case of GBS sepsis was reported for each of the study groups. Screening, randomization, and follow-up are shown in Fig. 1.

## Discussion

Maternal colonization with GBS is the predominant risk factor for both early- and late-onset neonatal sepsis, causing significant morbidity and mortality worldwide^11^. Despite the protective effect of IAP, infants of mothers who were GBS colonized but received < 4 h of IAP are at risk of presenting with sepsis^12^. Our randomized, double-blinded, placebo-controlled trial sought to evaluate the potential of oral probiotics as biological agents to eradicate GBS. We found a discreet, but statistically insignificant difference in vaginal GBS colonization between women who received oral probiotics and those who did not.

Since the 1980s, it has been known that intrapartum administration of antibiotics is one of the most effective measures to reduce the risk of early-onset GBS sepsis^13,14^. However, there is a substantial gap between optimal and actual IAP to prevent maternal-to-neonatal GBS transmission^15^. Newborns exposed to IAP show higher incidences of thrush and allergic sensitivities^11,16^. In addition, IAP involves the possibility of maternal anaphylaxis^17^, and increasing rates of resistance to antibiotic agents necessitate alternatives to conventional IAP^18^.

Administration of lactobacilli is an interesting alternative approach for the management of GBS colonization in pregnant women. *Lactobacillus (L.) rhamnosus* is known to produce the bacteriocin Lactocin160, which inhibits the growth of many bacteria—e.g. *Gardnerella vaginalis*. Lactocin160 destroys the cell membrane and induces adenosine triphosphate (ATP) leakage, killing bacterial vaginosis (BV) associated microorganisms while leaving the healthy vaginal microflora intact. Apart from that, lactobacilli produce hydrogen peroxide, which restores the normal vaginal microflora, compete with other bacteria for adherence to epithelial cells, and produce micromic acid, which leads to the acidification of the vaginal milieu^19^. Lipoteichoic acid (LTA), located on the cell surface of various bacteria, is known to stimulate the release of immune mediators, showing improved anti-inflammatory activity when being removed or substituted^20,21^. Knowledge of these mechanisms led to the hypothesis that lactobacilli might also eradicate microorganisms other than those that are BV-associated. In 2014, Hanson et al.^22^ reported that prenatal intake of probiotics had the potential to reduce GBS colonization and suggested that controlled clinical trials should be performed to prove this hypothesis.

In an experimental murine model, seven doses of 10^8^ viable cells of *L. reuteri* CRL1324 could effectively reduce the number of GBS^23^. Compared to four doses, seven doses caused a slight increase in vaginal colonization with *L. reuteri* CRL1324, and reduced murine vaginal pH compared to control mice. The authors postulated that lactobacilli could be considered as biological agents to reduce infections caused by GBS^23^. In the present study, we chose oral administration of a probiotic agent comprising four viable lactobacilli strains, given that we were able to demonstrate in our previous work that there is a high degree of overlap between vaginal and rectal strains of lactobacilli, and that lactobacilli strains colonize the mouth, vagina, and rectum simultaneously^24^. The probiotic selected has been proven to transmigrate from the intestinal lumen into the vaginal area, acting against pathogenic microorganisms in the microbial ecosystem through a barrier effect, so that symptoms related to dysbiosis and recurrent infections could be relieved or even prevented^25–27^.

After a 14-day treatment course with a four-strain probiotic, follow-up smears of women in the verum group of our study showed slightly lower rates of GBS colonization compared to women who received the placebo, which is of particular interest as it describes our primary endpoint. The numbers would have suggested a benefit from probiotic treatment (63.6% vs. 77.8% persisting GBS positivity after treatment in the verum and placebo groups, respectively), but the difference was not statistically significant upon logistic regression analysis. In 2016, a study in Taiwan evaluated the potential of *L. reuteri* RC-14 and *L. rhamnosus* GR-1 to eradicate GBS, reporting a positive-to-negative conversion rate of 42.9% in the women who received the probiotics, which was statistically different from the placebo group^28^. Our findings could not confirm this earlier finding, although the difference between the two studies might have arisen from the small number of participants in the final analysis of our study data due to the high dropout rates, which could have made it difficult to find statistical significance. Apart from that, our findings could have been affected by the inhibition of autophagy in vaginal epithelial cells by GBS-induced hsp70 production, which is associated with GBS persistence^29^. Concurrently, alterations in components known to influence vaginal bacterial colonization or facilitate microbial passage to the upper genital tract also occur in relation to GBS carriage.

Although a diverse array of anaerobic and aerobic microorganisms form the vaginal microbiome, it is dominated by lactobacilli that colonize mucous membranes, compete with other microorganisms for adherence to the vaginal epithelium, produce antimicrobial compounds, and modulate the immune response^30^. It has previously been shown that the absence of lactobacilli during early pregnancy increases the risks of vaginal dysbiosis and preterm birth^7^. Our data could not show that the intake of probiotics decreased the risk of preterm birth, which we already anticipated, as the intake of probiotics was limited to the third trimester of pregnancy. Generally, we found neither beneficial nor detrimental effects of probiotics with regard to the observed obstetric outcomes. Of note, cigarette smoking was detected as a preventable risk factor that was associated with preterm birth, as is well described in the literature^31^. The fact that no patient in our cohort experienced GBS sepsis is reasonable in the light of the available literature^32,33^. It is well known that newborns born to GBS-positive mothers who received IAP show significantly lower sepsis rates, but the time-to-delivery and the number of received dosages has also a strong impact on this effect^34^. The prevalence of GBS colonization of infants born to colonized mothers decreases with increasing duration of IAP, from almost 50% within 1 h of IAP initiation, to 28% after 1–2 h, 2.9% after 2–5 h, and 1.2% after 4 h^35^. All women in our patient cohort received IAP within 3 h, which could explain why we did not observe any case of neonatal sepsis. Nevertheless, the association between the period from IAP initiation and neonatal sepsis underlines the rationale of protective measures to a priori reduce GBS colonization rates.

Despite the generally high quality of our study design, there are some limitations. Most importantly, the number of patients that were finally randomized was lower than we had initially anticipated to achieve adequate power. Moreover, we analyzed a relatively small sample with available follow-up smears. The dropout rate of 36.7% in our cohort is far too high; most of the dropouts were caused by birth prior to the scheduled follow-up smear. This limitation might have arisen from the fact that our tertiary referral center primarily serves high-risk pregnancies that are more likely to result in premature or unscheduled birth. Indeed, we could have collected the follow-up smears immediately before birth, but this would have introduced bias to our data because treatment would have been still ongoing in many such cases. It is also possible that GBS testing yielded false positive or false negative results; quantitative maternal cultures would have been of value. In our study, intrapartum antibiotics were administered to GBS-colonized mothers, and the study design did therefore not reliably allow evaluating the impact of maternal probiotics on infant colonization and neonatal outcomes. Probiotic intake for 14 days might have also been too short to find a significant difference in GBS colonization between the verum and placebo groups, which could explain the trend toward a reduced colonization rate after probiotic intake, which we found in our study.

In conclusion, the present study neither confirmed, nor refuted the hypothesis that oral probiotics have the potential to eradicate GBS during pregnancy. We found a trend toward lower rates of GBS persistence in women who received probiotics, but this trend was not statistically significant. Further studies with higher dosages and longer duration of intake, as well as those with different combinations of probiotic strains, are necessary to prove our hypothesis. Probiotics might also reduce the risk of neonatal sepsis without eradicating GBS from the maternal genital tract.

## Methods

### Ethical considerations

This randomized, double-blinded, placebo-controlled trial was conducted in accordance with the Declaration of Helsinki and good clinical practice guidelines. The study protocol was approved by the Ethics Committee of the Medical University of Vienna (reference number 1001/2017). Study participants provided informed consent prior to randomization and all data were anonymized and de-identified before analyses. The trial was registered in a publically accessible trial registry (ClinicalTrials.gov-ID: NCT03008421), available under the following URL: https://clinicaltrials.gov/ct2/show/NCT03008421 (date of registration: 02/01/2017; Date of first enrollment: 01/06/2018).

### Study setting

The study was conducted at the Medical University of Vienna, Vienna General Hospital (Vienna, Austria), a 2200-bed tertiary referral center that provides medical attendance to about 99,000 inpatients and 500,000 outpatients per year. The Department of Obstetrics and Gynecology is specialized in high-risk obstetrics and performed up to 3000 deliveries per year. Pregnancy care in this department included a prenatal consultation between 10–0/7 and 16–0/7 gestational weeks, during which women registered for a planned delivery and underwent routine screening for vaginal infections by Gram-staining of the vaginal flora^36^. In case of an infection, a follow-up smear was performed after 4–6 weeks. Routine pregnancy care was undertaken at outpatient gynecologist offices, following the Austrian government’s welfare program as documented in the official mother–child booklet. This is a nationwide pregnancy care program that is used as a health precaution for women and fetuses, including obligatory examinations at predetermined points.

### Identification and screening

Women with singleton pregnancies, without imminent miscarriage, and without recent antibiotic or probiotic treatment, were considered eligible for study inclusion. After informed consent, participants were asked to present for GBS screening between 32–6/7 and 36–6/7 gestational weeks. During this first visit, a CDC-recommended combined vaginal-to-rectal swab was collected from the *introitus vaginae* and anorectum^37^. The combined incubation is known to increase the detection rate of GBS by up to 30%^38^. Sterile swabs were obtained after vaginal fluid collection from the lateral vaginal wall, anterior and posterior *fornix vaginae*, followed by insertion into the anal sphincter, using the ESwab collection and transport system (Copan Diagnostics, Murrieta, CA, USA). Specimens were analyzed at the Division of Clinical Microbiology of the Department of Laboratory Medicine, Medical University of Vienna (Vienna, Austria). For culture, both a selective solid medium for gram-positive bacteria (Columbia CNA agar with 5% sheep blood Improved II, Becton Dickinson, Heidelberg, Germany) and a selective enrichment broth (Todd Hewitt Broth & Antibiotic, Bio Merieux, Marcy l'Etoile, France) were inoculated, followed by incubation for 18–24 h at 37 °C under aerobic conditions. In case of growth only in the enrichment broth, a subculture using the selective solid medium for another 18–24 h was performed as mentioned above. For pathogen identification to the species level suspicious colonies were analyzed by matrix-assisted laser desorption ionization-time of flight mass spectrometry (MALDI-TOF MS) on a microflex instrument (Bruker Daltonics, Bremen, Germany)^33,39^. Women were enrolled for the study following informed consent, receiving either verum or placebo. A consecutive swab was collected from each participant 3–4 weeks after the initial visit. Here, ESwabs swabs were obtained and inoculated as previously described^16^. In cases of persistent GBS colonization, participants were informed and the result was documented both in the mother–child booklet and the in-house perinatal chart of the patient. This procedure was chosen to ensure intrapartum antibiotic treatment, as recommended by our guidelines^11^.

### Inclusion and exclusion criteria

Women with singleton pregnancies, who were found to be GBS-positive at the first study visit, without any signs of ongoing infections or bleedings, were considered eligible for the study. In contrast, women with multiple pregnancies, as well as those with BV, trichomoniasis, history of and/or ongoing vaginal bleedings, recent antibiotic, and/or probiotic use (i.e., within 4 weeks prior to randomization), were considered not eligible. BV was defined as a Nugent score of 7–10 on Gram-stained smears. Women with BV had to be excluded from the study, as they would have had to be treated with antibiotics, which in turn might have influenced our results.

### Study medication

Women of the verum group received a patented dietary supplement comprising four viable *Lactobacillus* strains. Ingredients per capsule were as follows: 84 mg fructo-oligosaccharides; 0.2 billion *L. jensenii* Lbv116 100 B UFC/g (DSM 22566); 1 billion *L. crispatus* Lbv88 100 B UFC/g (DSM 22566); 1 billion *L. rhamnosus* Lbv96 100 B UFC/g (DSM 22560); 0.3 billion *L. gasseri* Lbv150 100 B UFC/g (DSM 22583); 60 mg corn starch; 3 mg magnesium stearate; 3 mg silicon dioxide; 73.56 mg hydroxypropyl-methylcellulose, and 1.43 mg E171 titanium dioxide coloring. The manufacturer issued a 'certificate of analysis' that guaranteed adequate quality control, which is available upon reasonable request from the corresponding author.

### Randomization and study groups

Participants were randomized using Randomizer (Institute for Medical Informatics, Statistics and Documentation, Medical University of Graz), a web-based patient randomization service for clinical trials. In the verum group, administration of the probiotic supplement included the twice-daily (morning/evening) oral intake for a total of 14 days. In the placebo group, women received an oral potato-maltodextrin-based placebo that was visually indiscernible from the treatment and was likewise administered twice daily for 14 days. Patients, investigators, and microbiological staff were blinded to study group assignment. In cases of insufficient intake of the study medication, any use of antibiotic or probiotic drugs during the study period, or delivery prior to the follow-up visit, subjects were excluded from data analyses. Intake of the study medication was considered sufficient in patients that did not exceed a total of 3 out of 14 days treatment discontinuation, which was monitored by the return of the packages after the final study visit.

### Study endpoints

Colonization with GBS at the follow-up visit was considered to be the primary endpoint. Secondary endpoints were the following: neonatal sepsis, gestational age at delivery (recorded as term delivery at or after 37 gestational weeks), neonatal birthweight (defined as the weight at the time of delivery), and mode of delivery (i.e., vaginal birth, including instrumental vaginal birth versus C-section). Neonatal sepsis was defined as early- or late-onset sepsis, based on the age at onset, with bacteremia (i.e., blood culture positive) at less than 7 days in term infants as early-onset, or later than 7 days until 3 months as late-onset sepsis.

### Sample size calculation

The power for the present study was calculated using the dichotomized endpoint “recovery yes/no”, where recovery “yes” was defined as the absence of GBS colonization at the follow-up visit. For sample size calculation, we assumed that 43% of women in the verum group would achieve the primary endpoint (i.e., recovery to the GBS negative state). This assumption was based on previously published clinical and preclinical data^23,28,40^. We expected an 18% recovery rate in the placebo group. In order to achieve a 90% power to detect the group difference, and considering a 10% drop-out rate, we would have needed to screen 1040 patients to be able to randomize 70 patients per group, at an alpha level of 0.05. However, after screening 1058 patients, we were only able to randomize a total of 82 patients (41 patients per group), leading to a power of 70% to detect the originally assumed group difference.

### Statistical analysis

Continuous variables were summarized using mean, standard deviation, median, and quartiles as well as minimum and maximum. Categorical variables were summarized using absolute and percent values. Descriptive statistics are shown for all patients and separately for the placebo and verum groups. Baseline variables were compared between the groups using the *t*-test or Wilcoxon test for continuous variables, and the Fisher's exact test for categorical variables. To investigate the influence of study group (verum or placebo) on GBS colonization at the primary endpoint (follow-up visit), a univariate logistic regression model was performed first. Univariate logistic regression models for confounding factors, e.g. smoking status or maternal age, were also calculated. If more than one factor showed a significant influence on GBS colonization in the univariate regression model (*p* < 0.05), multivariate logistic regression models accounting for all significant confounding factors in the univariate model were calculated. Similar analyses were performed for the secondary endpoints. Linear regression models were calculated for birth weight. History of preterm birth, Apgar < 7 and candidosis at enrollment could not be considered as confounding factors due to the low number of observed cases. Data was extracted from obstetric databases, patient charts, and microbiologic reports, using the PIA Fetal Database, version 5.6.16.917 (General Electric Company, GE Viewpoint, Munich, Germany). Statistical calculations were performed using the R-Project for Statistical Computing, version 3.3.3 (R Development Core Team, Boston, MA, USA) by an investigator at the Institute for Medical Statistics (IMS), Center of Medical Statistics, Informatics and Intelligent Systems at the Medical University of Vienna.

## Data Availability

The data are available on request to the corresponding author.
